# Complete and Assembled Genome Sequence of *Vagococcus teuberi* DSM 21459^T^, a Novel Species Isolated from Fermented Cow Milk in Mali

**DOI:** 10.1128/genomeA.01514-16

**Published:** 2017-01-26

**Authors:** Marc J. A. Stevens, Raffael C. Inglin, Leo Meile

**Affiliations:** Laboratory of Food Biotechnology, Institute of Food, Nutrition and Health, ETH Zurich, Zurich, Switzerland

## Abstract

The genome of *Vagococcus teuberi* DSM 21459^T^, a strain isolated from Malian fermented milk, was sequenced using single-molecule real-time sequencing. The genome of *V. teuberi* DSM 21459^T^ is the first sequenced genome of this novel species and the second genome among the genus *Vagococcus*.

## GENOME ANNOUNCEMENT

Lactic acid bacteria belonging to the genus* Vagococcus* mostly originate from aquatic or animal environments (1). A lactic acid bacterium that was recently identified as a predominant bacterium in the spontaneously fermented cow milk product fènè from Mali (2) turned out to be the novel species *Vagococcus teuberi* (3). From the genus *Vagococcus*, only the draft genome of *Vagococcus lutrae* strain LBD1 is available so far (4). The presence of high numbers of this particular *Vagococcus* strain in fermented milk products has raised questions about the role of *V. teuberi* in fermented milk. Therefore, we sequenced the genome of the type strain DSM 21459 to acquire more information on its potential functions.

Genomic DNA was isolated using the Wizard genomic DNA purification kit (Promega, Dübendorf, Switzerland) using enzymatic cell lysis prior to purification, as described previously (5). The genome was sequenced using single-molecule real-time cells on a PacBio RS II (Pacific Biosciences, Menlo Park, CA, USA) platform at the Functional Genomics Center Zurich (Zurich, Switzerland). The output was 91,269 reads with an average length of 11,310 bp, resulting in 1,032,279,404 bp and a coverage of 475×. The reads were corrected using the “correct pacbio reads” tool in the Genome Finishing Module of CLC Genomics Workbench version 8.0, and the corrected reads were used for a *de novo* assembly using CLC Genomics Workbench with standard settings. After *de novo* assembly, contigs were joined using the “join contigs” tool in the Genome Finishing Module of CLC Genomics Workbench version 8.0. The assembly resulted in two contigs—one for the chromosome and one for a 15-kb plasmid.

The genome was annotated by the NCBI Prokaryotic Genome Automatic Annotation Pipeline. The genome consists of a 2,169,064-bp chromosome and a 15-kb plasmid. It comprises 59 tRNA genes and three rRNA operons. The G+C content of the genome is 34.1%, and a total of 1,973 protein coding sequences were predicted.

Data from this genome can now be used to elucidate whether *V. teuberi* plays a role as a lactic acid bacterium starter culture, a novel function which has not been attributed so far to the *Vagococcus* genus.

### Accession number(s).

Sequence and annotation data of the complete genome of *V. teuberi* strain DSM 21459^T^ are deposited in the GenBank database under the accession numbers CP017267 for the genome and CP017268 for the plasmid.
